# De Novo Self-Assembling Peptides Mediate the Conversion of Temozolomide and Delivery of a Model Drug into Glioblastoma Multiforme Cells

**DOI:** 10.3390/biomedicines10092164

**Published:** 2022-09-02

**Authors:** Megan Pitz, Margaret Elpers, Alexandra Nukovic, Sarah Wilde, Arica Jordan Gregory, Angela Alexander-Bryant

**Affiliations:** Nanobiotechnology Lab, Department of Bioengineering, Clemson University, Clemson, SC 29634, USA

**Keywords:** self-assembling peptides, temozolomide, glioblastoma multiforme

## Abstract

Glioblastoma multiforme (GBM) is the most aggressive central nervous system tumor, and standard treatment, including surgical resection, radiation, and chemotherapy, has not significantly improved patient outcomes over the last 20 years. Temozolomide (TMZ), the prodrug most commonly used to treat GBM, must pass the blood–brain barrier and requires a basic pH to convert to its active form. Due to these barriers, less than 30% of orally delivered TMZ reaches the central nervous system and becomes bioactive. In this work, we have developed a novel biomaterial delivery system to convert TMZ to its active form and that shows promise for intracellular TMZ delivery. Self-assembling peptides were characterized under several different assembly conditions and evaluated for TMZ loading and conversion. Both solvent and method of assembly were found to affect the supramolecular and secondary structure of peptide assemblies. Additionally, as peptides degraded in phosphate-buffered saline, TMZ was rapidly converted to its active form. This work demonstrates that peptide-based drug delivery systems can effectively create a local stimulus during drug delivery while remaining biocompatible. This principle could be used in many future biomedical applications in addition to cancer treatment, such as wound healing and regenerative medicine.

## 1. Introduction

Glioblastoma multiforme (GBM) is a grade 4 glioma tumor that develops in glial cells and accounts for 46.6% of all malignant central nervous system tumors [1]. GBM is the most aggressive central nervous system tumor due to the overproduction of vascular endothelial growth factor and the infiltrative tendrils of cancerous cells, which grow outward into healthy brain tissue [2]. GBM tumors are typically found in areas of the brain controlling speech, motor function, and the senses and are therefore especially difficult to treat effectively. The median survival time for patients diagnosed with glioblastoma multiforme is 12 to 15 months, and the five-year survival rate is 5.6% with standard treatment [2,3].

Standard treatment for GBM is a combination of surgical resection, radiation therapy, and chemotherapy [4]. However, surgery and radiation are often ineffective due to the infiltrative nature of these tumors, and surgery is sometimes impossible due to the tumor’s location in the brain. Temozolomide (TMZ) is the most commonly used chemotherapeutic to treat patients with GBM and is delivered orally [4]. TMZ initiates cell death via methylation of guanine residues at the O6 position, which creates a base pair mismatch and leads to apoptosis [5]. TMZ is a pH-sensitive hydrophobic prodrug that remains stable under acidic conditions. Upon exposure to physiological blood pH of around 7.4, conversion to the active form occurs via hydrolysis [6]; TMZ first converts to metabolite 3-methyl-(triazen-1-yl)imidazole-4-car-boxamide (MTIC) and then to 5-amino-imidazole-4-carboxamide (AIC) and a methyldiazonium cation [7]. The methyldiazonium cation is the active agent that methylates DNA, leading to cell apoptosis [7]. Therefore, the efficacy of TMZ is reliant on conversion of the drug once it leaves the stomach; however, recent studies have found that TMZ requires a more basic pH (>7.5) to rapidly and fully convert to its active form [5]. Furthermore, the active form of TMZ is not capable of crossing the blood–brain barrier (BBB) [6]. Due to these obstacles, only about 30% of orally delivered TMZ reaches the central nervous system [8]. Additionally, the plasma half-life of TMZ is 1.8 h, similar to other oral chemotherapies, such as capecitabine [6,9,10]; oral chemotherapies with short half-lives require more frequent dosing and therefore result in reduced patient compliance [11]. Thus, insufficient tumor accumulation, lack of conversion of TMZ, and frequent dosing requirements result in low efficacy when administered orally in standard chemotherapeutic treatment.

To bypass the BBB, local delivery systems have been developed to deliver therapeutics to treat GBM. The only FDA-approved local delivery system for GBM is the Gliadel^®^ Wafer, a biodegradable polyanhydride copolymer wafer loaded with the anticancer agent carmustine (BCNU) [12]. The wafer has been shown to prolong the survival of some GBM patients by 3.3 months compared to patients who did not receive the wafer as treatment; however, the toxicity of the wafer induces side effects such as seizures, cerebral edemas, and infections [7,12]. Other studies have revealed no significant increase in survival after insertion of Gliadel^®^ wafers [13]. Another drawback of this local delivery system is that it is only applicable for surgically resectable GBM tumors. Therefore, there is a need for an effective treatment strategy that enhances chemotherapeutic efficacy, successfully crosses or bypasses the BBB, and can be used to treat both resectable and nonresectable GBM tumors.

This study explores peptide self-assemblies as an injectable local drug delivery vehicle that may be injected directly into the brain and bypass the BBB. Self-assembling peptide hydrogels are widely used in drug delivery because they can form a variety of nanostructures. Compared to polymer-based drug delivery systems, peptide-based systems are generally more biocompatible [14]. Short peptides, often consisting of alternating hydrophobic and hydrophilic regions, self-assemble in aqueous solutions to form nanoparticle, nanotube, or nanofiber structures with beta-sheet or alpha helix secondary structures [14,15,16]. Self-assembly occurs due to a combination of hydrogen bonding, Van der Waals forces, hydrophobic interactions, and ionic bonding between oppositely charged amino acids [17].

This study compares five peptide sequences as potential drug delivery systems for TMZ under three assembly conditions. Three peptide formulations, namely ALK1, ALK2, and ALK3, are composed of varying amounts of alanine (A), glutamic acid (R), and lysine (K), each with different ratios of hydrophilic and hydrophobic amino acids to promote self-assembly. This series of peptides follows an amphiphilic design, with a basic lysine tail of varying lengths. Two peptide formulations, namely RAE and RAEF, use alanine (A), glutamic acid (E), arginine (R), and phenylalanine (F) and are based on alternating sequences commonly used in peptide delivery systems, such as RADA16 and EAK16 [14,18,19,20,21]. Peptides were characterized using circular dichroism, scanning electron microscopy (SEM), and rheological analysis. We also examined the ability of the peptides to load and convert TMZ to its active form and to deliver a model hydrophobic drug into GBM cells. It is hypothesized that as the peptides self-assemble, TMZ will load into the hydrophobic regions consisting of alanine and glutamic acid and remain stable while in contact with acidic residues. As the peptide assemblies degrade, TMZ will be exposed to basic lysine or arginine residues and converted to its active form. Conversion of prodrugs using material degradation products has not been previously explored, and the successful realization of this goal could advance applications of prodrug-based delivery systems.

## 2. Materials and Methods

### 2.1. Materials

Peptides were synthesized using Fmoc synthesis by Genscript (Piscataway, NJ, USA) and LifeTein (Somerset, NJ, USA) and purified using high-performance liquid chromatography (>95% purity). All peptides were synthesized with N-terminal acetylation and C-terminal amidation. Peptide sequences are shown in Table 1. LN-18 human glioblastoma cells were obtained from American Type Culture Collection (ATCC, Manassas, VA, USA) and cultured according to ATCC guidelines. Temozolomide (TMZ) was obtained from Selleck Chemical (Houston, TX, USA). Dulbecco’s Modified Eagle’s Medium (DMEM), fetal bovine serum (FBS), and antibiotic/antimycotic solution were purchased from Corning Inc. (Corning, NY, USA). Hexamethyldisilazane (HMDS) was purchased from Sigma-Aldrich (St. Louis, MO, USA). CellTiter 96 Aqueous One Solution Cell Proliferation Assay was purchased from Promega (Madison, WI, USA). Coumarin-6 (C6) fluorescent dye was purchased from Thermo Scientific (Waltham, MA, USA).

### 2.2. Materials Characterization

#### 2.2.1. Peptide Self-Assembly

Lyophilized peptides from GenScript and LifeTein were rehydrated in ddH_2_O to create 10 mg/mL stock solutions which were frozen at −60 °C until use. To initiate self-assembly, two methods were used: method 1 is a film dehydration method in which peptide stocks were aliquoted into Eppendorf tubes, 90% EtOH was added for a total volume of 100 µL, and solutions were allowed to completely evaporate overnight, creating a peptide film on the bottom of the tube. The peptide film was then rehydrated with ddH_2_O or phosphate-buffered saline (PBS) to a final concentration of 1 mg/mL and shaken on a shake plate for 4 h to initiate assembly. Method 2 is a simple incubation assembly in which stocks were thawed and diluted with PBS to a final peptide concentration of 1 mg/mL. Peptide solutions were allowed to self-assemble for 24 h at room temperature.

#### 2.2.2. Circular Dichroism

Peptide assemblies were loaded into a 1 cm cuvette, and CD spectra were recorded from 190–300 nm as an average of four accumulations at room temperature on a Jasco 810 Polarimeter (Jasco Corp., Tokyo, Japan). Samples were diluted as necessary to record full spectra. A reference spectrum of each solvent was recorded and subtracted from each spectrum. Data were analyzed using DichroWeb online software with the K2D algorithm [22,23].

#### 2.2.3. SEM

Peptide assemblies were dried using a well-known EtOH:HMDS method [24]; briefly, peptide assemblies were centrifuged into a pellet, and the supernatant was removed and replaced with EtOH. Assemblies were then vortexed briefly to reconstitute in EtOH. This process was repeated three times with EtOH and three times with HMDS. Peptide assemblies were then dropped onto silicon wafers and dehydrated overnight at room temperature. Wafers containing dried peptide assemblies were sputter-coated with platinum before imaging with a Hitachi Regulus 8230 (Hitachi, Tokyo, Japan).

#### 2.2.4. Rheology

Rheological analysis was used to determine the shear-dependent viscosity of peptide self-assemblies using an Anton Paar Modular Compact Rheometer 302e (Anton Paar, Graz, Austria). Briefly, peptide assemblies were placed on a 60 mm cone plate geometry. Linear ramping shear was applied from 0.1–300 s^−1^. Viscosity was measured using the Rheocompass software (Anton Paar, Graz, Austria).

#### 2.2.5. Drug Loading and Conversion

To examine loading efficiency, TMZ was loaded into each peptide formulation at varying concentrations. Loading was achieved differently for each assembly method; to load the film dehydration peptide assemblies, TMZ solution was added before evaporation to allow incorporation into the peptide film. Before rehydration, the film was washed three times with ddH_2_O to remove unloaded TMZ. To load the incubation peptide assemblies, stock peptides and TMZ were co-assembled in water. After allowing 24 h for self-assembly, the assembled peptides were spin-filtered to remove unloaded TMZ. The loaded peptide assemblies were then resuspended in either ddH_2_O or PBS. TMZ concentration in the loaded assemblies was then measured using UV–vis spectrophotometry on a Synergy plate reader (BioTek, Winooski, VT, USA). Loading efficiency was calculated using Equation (1):

(1) $$EE\left(\%\right)=\frac{W_{loaded\;drug}}{W_{total\;drug}}\times 100\%,$$

where $W_{total\;drug}$ is the concentration of TMZ initially added to the solution, and $W_{loaded\;drug}$ is the concentration of TMZ in solution after peptide assembly and removal of excess drug. TMZ conversion over time was measured using UV–vis spectrophotometry on the same peptide assemblies over six days. Both TMZ and AIC concentrations were measured to determine percent drug conversion. For cellular uptake experiments, coumarin-6 fluorescent dye was loaded in the same manner as TMZ for all peptide formulations.

### 2.3. Cell Studies

#### 2.3.1. MTS

MTS experiments were conducted to determine cell viability. Briefly, LN-18 human glioblastoma cells were seeded into a 96-well plate (10 k/well) to achieve 50–60% confluence and allowed to attach overnight. The following day, cells were incubated with peptide assemblies for 48 h, after which CellTiter MTS reagent was applied to each well and incubated for 3 h according to the manufacturer’s protocol. A Synergy UV–vis plate reader (BioTek, Winooski, VT, USA) was used to measure the absorbance of the solution at a wavelength of 490 nm. Untreated cells (negative control) were used to normalize viability for all MTS experiments.

#### 2.3.2. Cellular Uptake

LN-18 cells were seeded in a 24-well plate (25 k/well) and allowed to attach overnight. The following day, cells were treated with peptide assemblies loaded with the fluorescent dye coumarin-6 (C6). After 4 or 24 h, cells were first live imaged using an EVOS FL fluorescent microscope (Life Technologies Corporation, Bothell, DC, USA). Briefly, NucBlue stain was applied to cells for 15 min, then removed, and replaced with 1×PBS. Cells were imaged on the EVOS DAPI laser to identify cell nuclei and on the EVOS green laser to identify C6. After imaging, cells were trypsinized and analyzed for a positive fluorescent signal with the BL1 laser line (530/30) using the Attune NxT flow cytometer (Invitrogen, Waltham, MA, USA).

### 2.4. Statistical Analysis

Statistical analyses were carried out by one-way ANOVA followed by Tukey’s post hoc test (GraphPad Prism 8, San Diego, CA). Values were expressed as mean ± SEM of three independent experiments; p-values < 0.05 were considered statistically significant.

## 3. Results and Discussion

### 3.1. Characterization of Assemblies Formed via Film Dehydration Method

The secondary structure of peptide self-assemblies formed using the film dehydration method was examined primarily using circular dichroism (CD), shown in Table 2. High percentages of irregular structures (40–60%) and low percentages of alpha-helical structures (0–10%) were detected for all formulations except for ALK1. Alanine and glutamic acid have the highest propensity to form alpha-helical structures, while continuous sequences of charged residues break up alpha helices [25]. ALK1 has the smallest lysine tail and the most alanine and glutamic acid residues; thus, it was expected that ALK1 formed the highest percentage of alpha-helical secondary structures. Additionally, peptides assembled in water exhibited more beta-sheet formation than peptides assembled in PBS. Salts are known to act as kosmotropes or chaotropes in protein and peptide folding, causing differences in secondary structure between solvents [16]. Peptide hydrogels previously characterized in the literature, such as RADA16 and EAK16, exhibit beta-sheet secondary structures; therefore, beta-sheet structures may be indicative of hydrogel-like supramolecular assemblies [26,27].

**Figure 1:**
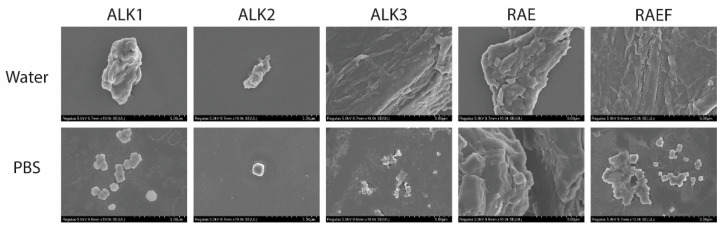
Peptides were prepared using film dehydration and then resuspended in either water or PBS. Samples were dried and sputter-coated with platinum before conducting SEM. All images were captured at a magnification of 10.0 k, and scale bars represent 5 µm.

SEM was used to analyze the supramolecular structure of peptide assemblies formed via film dehydration. Rehydration in water exhibited larger assemblies for almost all sequences (Figure 1). Films rehydrated in PBS appeared to form more globular and aggregated assemblies, all creating spherical structures except for RAE. Films rehydrated in water, especially ALK3, RAE, and RAEF, formed singular larger assemblies. ALK3 and RAE both appeared to assemble into fibrous structures in water. Additionally, RAE was the most consistent when comparing rehydration in water or PBS, forming large structures under both conditions. ALK1, ALK3, and RAEF peptides assembled in water drastically differed from structures assembled in PBS. Each of these sequences exhibited large, singular assemblies in water compared to small aggregated spherical structures in PBS. These results indicate that PBS acts as a chaotrope, interfering with peptide assembly and preventing elongation of supramolecular structures. The scales of assembly observed for ALK3, RAE, and RAEF in water and RAE in PBS are indicative of microgel formation. ALK1, ALK2, ALK3, and RAEF assemblies in PBS can be considered nanoparticles, which were slightly aggregated.

**Figure 2:**
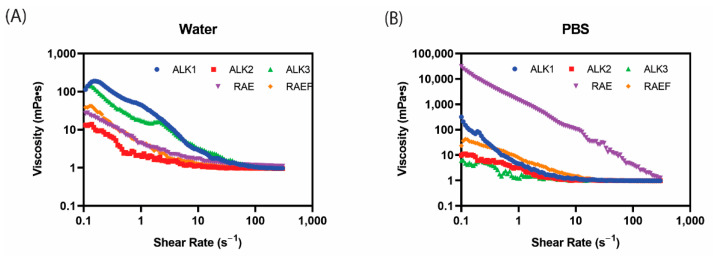
Peptides were prepared using film dehydration and then resuspended in either (**A**) water or (**B**) PBS. Samples were examined at shear rates from 1–300 Hz, and viscosity was plotted using Rheocompass.

Rheology was used to explore the rigidity and hydrogel formation of peptide assemblies via viscosity measurements. Increasing shear was applied to all peptide formulations, and the resulting viscosity was plotted as shown in Figure 2. For all peptides assembled in water and PBS, viscosity decreased with increasing shear rate, exhibiting shear-thinning behavior, which is ideal for application via injection. In water, ALK1 and ALK3 demonstrated the highest viscosity at lower shear rates, around 100 mPa·s. All formulations in water reach a viscosity of around 1 mPa·s by a shear rate of 100 s^−1^. In PBS, RAE exhibited a much higher viscosity compared to other peptide sequences at low shear rates. RAE assembled in PBS was the only film formulation that did not reach a viscosity of 1 mPa·s at a shear rate of 100 s^−1^ or lower. This result aligned with SEM imaging of RAE, which was the only sequence to form large supramolecular assemblies in PBS.

### 3.2. TMZ Loading and Conversion in Assemblies Formed via Film Dehydration Method

**Figure 3:**
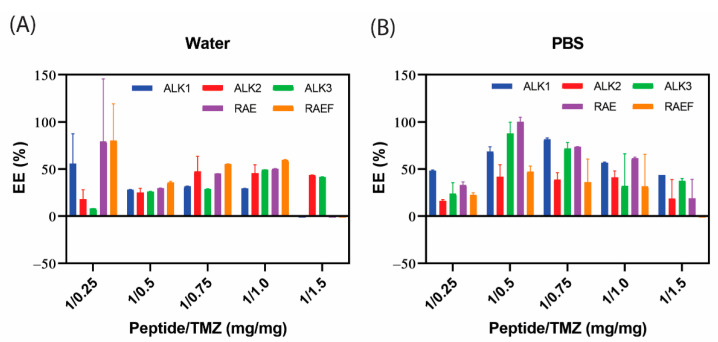
Peptides were prepared and loaded with TMZ using film dehydration and resuspended in either (**A**) water or (**B**) PBS. TMZ concentration was read using UV–vis spectrophotometry and plotted as encapsulation efficiency (%). Data are presented as mean ± SEM (N = 3).

TMZ loading into peptide assemblies formed by film dehydration was also examined. In water, peptide assemblies loaded between 50–100% of the applied drug, but the TMZ concentration with the highest loading efficiency varied among peptide sequences (Figure 3). In PBS, up to 200% loading is observed. Encapsulation efficiency over 100% may be due to the peptides increasing the absorbance reading. The large error indicates uneven loading in some peptide formulations; given that the peptide assemblies are not uniformly distributed in size as shown in SEM, loading may depend on each individual sample. For peptides rehydrated in water, the percent of loaded drug primarily increased with concentration. However, for peptides rehydrated in PBS, maximum loading was achieved at varying concentrations for each peptide formulation. This may be attributed to smaller, more irregular structure formation with peptides rehydrated in PBS.

**Figure 4:**
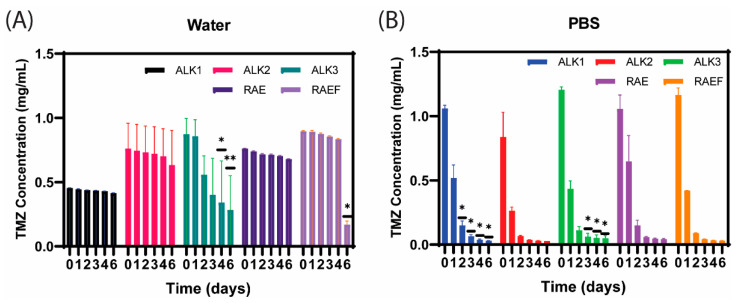
Peptides were prepared and loaded with TMZ using film dehydration and resuspended in either water (**A**) or PBS (**B**). TMZ concentration was measured from 0–6 days using UV–vis spectrophotometry. Data are presented as mean ± SEM (N = 3), where * *p* < 0.05 and ** *p* < 0.01 compared to time 0 (one-way ANOVA with Dunnett’s multiple comparisons test).

To determine whether the peptides effectively mediate TMZ conversion to its active form, TMZ concentration was measured in all peptide formulations over 6 days. For most peptide formulations rehydrated in water, very little TMZ conversion was observed over 6 days (Figure 4A). ALK3 was the only peptide that exhibited significant and rapid conversion in water. This suggests that the ALK1, ALK2, RAE, and RAEF peptides assembled in water remained stable and maintained the drug in an inactive state for at least 6 days. In contrast, all peptides assembled in PBS converted the loaded TMZ rapidly and completely after only 2 days (Figure 4B). The conversion profile of each formulation in PBS appeared very similar, with less than 15% of TMZ remaining inactive after 2 days. This can be attributed to the effects of PBS salts on peptide degradation. PBS salts mimic the in vivo environment and, at high concentrations, allow peptides and proteins to degrade [28]. Therefore, these results indicate that our peptide assemblies should rapidly degrade and convert TMZ to its active form after delivery in vivo.

Examination of results from film assemblies in water and PBS showed that while water-based formulations exhibit hydrogel structures, PBS formulations are more ideal for both loading and conversion of TMZ. For clinical applications, TMZ conversion is essential, making the PBS assemblies more viable. However, hydrogel structure is desirable for potential co-loading capabilities and for filling tumor resection cavities. Therefore, we also characterized peptide assemblies formed using a simple incubation method. Because better conversion of TMZ was achieved with PBS assemblies, subsequent synthesis of peptide assemblies was performed in PBS.

### 3.3. Characterization of Assemblies Formed via Incubation Method

**Figure 5:**
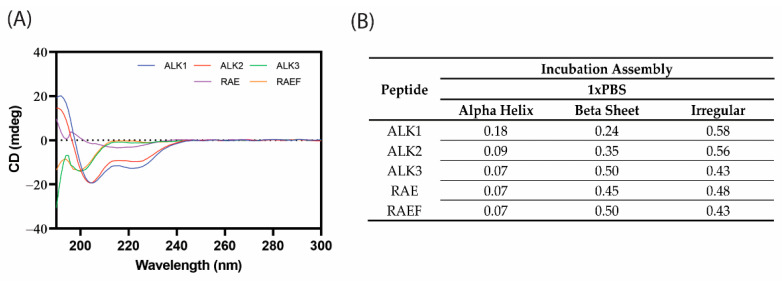
Peptides were assembled through incubation in PBS. Circular dichroism spectra were recorded from 190–300 nm (**A**). Spectra were analyzed on DichroWeb [22,23] using the K2D method to estimate secondary structure (**B**).

The secondary structure of assemblies formed through incubation in PBS was investigated using CD (Figure 5). The assembled peptides consisted of large percentages of beta-sheet secondary structures, especially in ALK3, RAE, and RAEF sequences. Similar to the film dehydration assemblies, there was little alpha-helix formation for all sequences except for ALK1 and about 50% irregular structures. Again, this was expected, as ALK1 contains the most alanine and glutamic acid residues, which have a propensity to form alpha helices. Because beta-sheets are associated with peptide hydrogel formation, high percentages of beta-sheet secondary structure were considered ideal. Overall, it appeared that the secondary structures formed using the incubation method did not significantly differ from the structures formed using film dehydration assembly although there was slightly more beta-sheet formation for ALK3, RAE, and RAEF.

**Figure 6:**
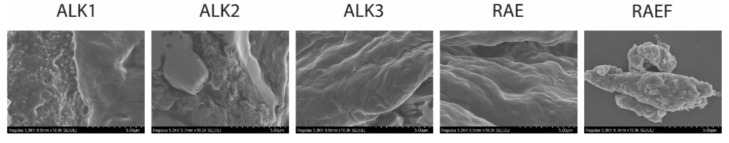
Peptides were assembled through incubation in PBS. Samples were dried and sputter-coated with platinum before conducting SEM. All images are captured at a magnification of 10.0 k, and scale bars represent 5 µm.

SEM imaging revealed that peptides incubated in PBS assembled into single, large supramolecular structures, as shown in Figure 6. There was an indication of beta-sheet-like structures in ALK3 and RAE, confirming CD results, while ALK1 and ALK2 appeared to form porous networks. RAEF appeared amorphous. Beta-sheet secondary structure and fibrous supramolecular structure are often observed in hydrogel formation for alternating peptide sequences. Therefore, ALK3 and RAE assembled via incubation in PBS were identified as microgels. The large scale of supramolecular assemblies (larger than 5 µm in diameter) for ALK1, ALK2, and RAEF also suggested microgel formation for these sequences. However, no gelation was visually observed during sample preparation. Therefore, rheology was necessary to characterize the rigidity of the supramolecular structures.

**Figure 7:**
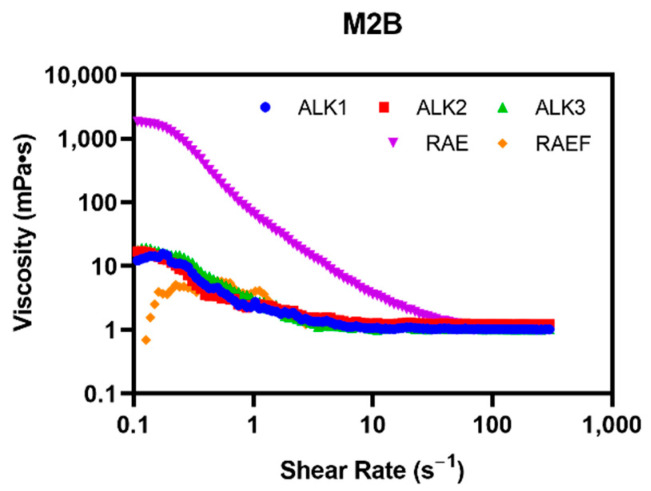
Peptides were prepared via incubation in PBS. Samples were examined at shear rates from 1–300 Hz and viscosity plotted using Rheocompass.

Rheology was explored to determine the viscosity of peptide assemblies and to determine if a hydrogel or microgel was formed. All peptide formulations assembled via incubation in PBS exhibited a higher viscosity at low shear rates and decreased viscosity as the shear rate increased (Figure 7). This is ideal for injections, which require shear-thinning materials. RAE showed the highest viscosity, larger than 1000 mPa·s, at low shear rates. This indicates that RAE may form the most networked or microgel-like structure compared to the other peptides with a viscosity similar to water at all shear rates.

### 3.4. TMZ Loading and Conversion in Assemblies Formed via Incubation Method

**Figure 8:**
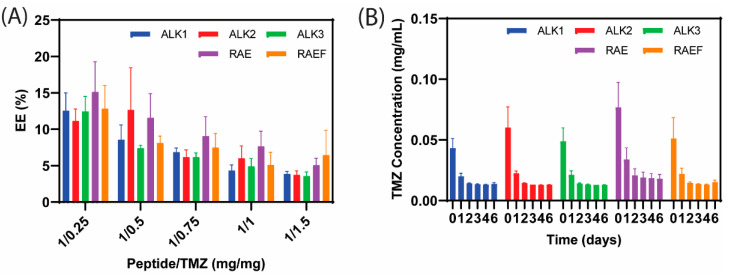
Peptides were prepared and loaded with TMZ using incubation assembly in PBS. TMZ concentration was read using UV–vis spectrophotometry, and encapsulation efficiency (EE) was calculated (**A**). TMZ conversion was measured over from 6 days (**B**) using UV-vis spectrophotometry. Data are presented as mean ± SEM (N = 3).

TMZ loading and conversion were evaluated in peptide assemblies formed through incubation in PBS using the same protocol as peptides assembled using thin film preparation. The encapsulation efficiency (EE) of TMZ in the PBS-incubated assemblies was below 10% for most peptide formulations (Figure 8A). Although EE was small, TMZ conversion was rapid, occurring after only one day, as shown in Figure 8B. The rapid conversion was likely due to the salts in PBS degrading peptide assemblies and triggering the dissociation of positively charged amino acids. As the peptide assemblies degrade, positive residues create a slightly basic environment, which assists in mediating pH-dependent TMZ conversion.

### 3.5. In Vitro Analysis of Peptide Biocompatiblity and Cellular Uptake

Characterization of all peptide formulations revealed that peptides assembled in PBS converted the most TMZ, while peptides assembled using thin film dehydration followed by rehydration in water exhibited both adequate drug loading and hydrogel structure compared to assemblies formed using the incubation method. Therefore, cell viability and cellular uptake were examined using the film dehydration method.

**Figure 9:**
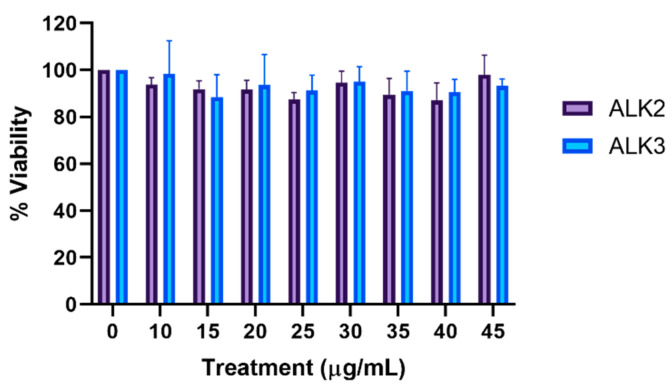
Peptides were prepared using film dehydration and rehydrated in water. Peptide assemblies were incubated with cells at varying concentrations for 48 h, followed by viability analysis via MTS assay. Untreated cells were treated as 100% viable. Results are presented as mean ± SEM (N = 3).

To determine peptide biocompatibility, we evaluated cellular viability. A large positive charge is associated with cytotoxicity [29]; thus, the biocompatibility of ALK2 and ALK3 were examined due to their large positive tails. Peptides assembled via the thin film method with rehydration in water were incubated with LN18 human glioblastoma cells in concentrations ranging from 0–45 µg/mL. MTS was used to determine cell viability compared to an untreated control, as shown in Figure 9. ALK2 and ALK3 did not significantly affect cell viability compared to untreated LN18 cells (one-way ANOVA with Dunnett’s multiple comparisons test) and were biocompatible at concentrations up to 45 µg/mL.

**Figure 10:**
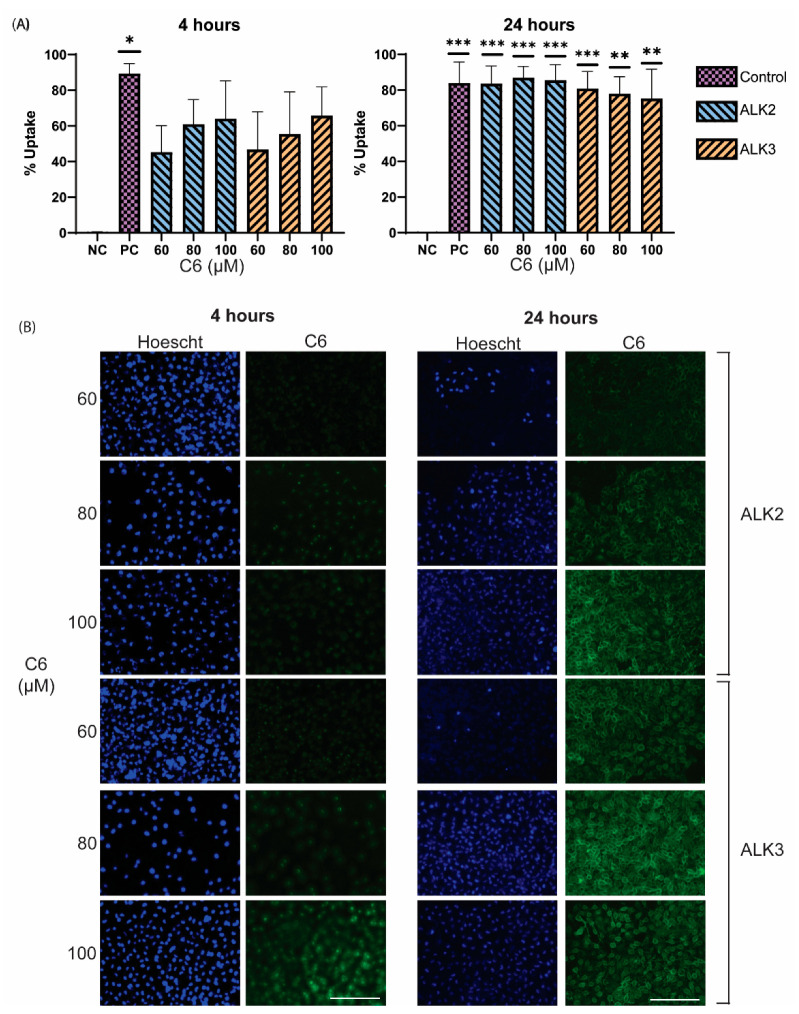
Peptides were mixed with 60, 80, or 100 µM C6 and then prepared using film dehydration and rehydrated in water. Peptide assemblies were incubated with cells for 4 or 24 h, followed by evaluation of cellular uptake using (**A**) flow cytometry and (**B**) fluorescent microscopy. Untreated cells were used as a negative control (NC), and 100 µM of C6 was used as a positive control (PC). Results are presented as mean ± SEM (N = 3), where * *p* < 0.05, ** *p* < 0.01, and *** *p* < 0.001 compared to NC (one-way ANOVA). Scale bar = 200 µm.

Finally, cellular uptake was explored to determine if peptide assemblies were viable for the intracellular delivery of therapeutics. C6, a fluorescent model drug, was loaded into ALK2 and ALK3 peptide assemblies using film dehydration in water. Both peptides mediated uptake of C6 into LN18 cells after 4 h, with between 40–60% uptake efficiency (Figure 10A). After 24 h, C6 uptake efficiency for both peptides increased to approximately 80%, similar to the efficiency of C6 alone (Figure 10B). As C6 dye is hydrophobic and readily crosses the cell membrane, it is promising that our peptide carriers achieved similar levels of cellular uptake. These results indicate that the peptide assemblies can effectively mediate the transport of their cargo across the cell membrane and demonstrates the potential of the peptides for the delivery of TMZ.

## 4. Conclusions

Assembly method and solvent have a pronounced effect on the secondary and supramolecular structures of peptide self-assemblies. While many formulations using the film dehydration method resulted in smaller, globular assemblies, the incubation method resulted in larger, more macro-scale structures for all sequences. The secondary structure was largely dependent on the solvent, with assemblies formed in water exhibiting more beta-sheet structure than PBS-based assemblies.

Drug interactions revealed that TMZ could effectively load into peptide assemblies up to 1 mg/mL. ALK2 and ALK3 peptides rapidly and completely converted loaded TMZ when peptides were degraded in PBS. This result is promising because PBS salts more accurately mimic the in vivo salt environment, indicating that peptide degradation and drug conversion will occur similarly in vivo.

Cellular interactions confirmed the biocompatibility of peptide assemblies, as treatment with unloaded peptides did not significantly affect cell viability. Additionally, peptide assemblies effectively mediated cellular uptake, indicating that these assemblies may be ideal vehicles for TMZ delivery.

Future work should determine the effect of drug-loaded peptides in vitro and in vivo. Because solvent and assembly method have a large impact on the supramolecular structure, additional solvents could be included in future analysis. Based on the results shown in this work, exploring the degradation of water-based peptide assemblies in various degradation environments could be a revealing next step.

## Figures and Tables

**Table 1:** Peptide names and sequences. Amino acid single letter abbreviations are used: alanine (A), glutamic acid (E), lysine (K), arginine (R), and phenylalanine (F).

Peptide Name	Sequence
ALK1	AEAEAEAEAEAEKKKK
ALK2	AEAEAEAEKKKKKKKK
ALK3	AEAEKKKKKKKKKKKK
RAE	RARAEARARAEARARAEA
RAEF	RRAEARRAFARRAEA

**Table 2:** Peptides were prepared using the film dehydration method and resuspended with water or PBS. Circular dichroism spectra were evaluated from 190–300 nm. Spectra were analyzed on DichroWeb [22,23] using the K2D method to estimate secondary structure.

Peptide	Film Dehydration					
	Water			1×PBS		
	Alpha Helix	Beta Sheet	Irregular	Alpha Helix	Beta Sheet	Irregular
ALK1	0.12	0.34	0.54	0.10	0.41	0.49
ALK2	0.04	0.48	0.48	0.09	0.37	0.54
ALK3	0.09	0.47	0.44	0.05	0.35	0.60
RAE	0.05	0.47	0.48	0.04	0.48	0.48
RAEF	0.05	0.47	0.48	0.05	0.47	0.48

## Data Availability

Data are contained within the article or supplementary material.
